# Clinical outcomes of intraluminal Iodine-125 seed strand brachytherapy and percutaneous nephrostomy in patients with ureteral carcinoma

**DOI:** 10.1186/s12885-023-10921-3

**Published:** 2023-06-06

**Authors:** Yonghua Bi, Dechao Jiao, Jianhao Zhang, Yang Wang, Mengdan Su, Jianzhuang Ren, Xinwei Han

**Affiliations:** Department of Interventional Radiology, the First Affiliated Hospital of Zhengzhou University, No.1, East Jian She Road, 450052 Zhengzhou, China

**Keywords:** Iodine-125 seed strand, Intraluminal brachytherapy, Ureteral carcinoma, Nephrostomy, Renal pelvis

## Abstract

**Background:**

We aimed to evaluate the safety and efficacy of intraluminal iodine-125 seed strand brachytherapy and percutaneous nephrostomy in patients with ureteral carcinoma.

**Methods:**

From January 2014 to January 2023, 48 patients with ureteral cancer not suitable for surgical resection were enrolled. Iodine-125 seed strand was inserted in 26 patients under c-arm CT and fluoroscopic guidance (Group A), and 22 patients underwent percutaneous nephrostomy without seed strand (Group B). The clinical outcomes (technical success rate, tumor sizes, hydronephrosis Girignon grade, complications, objective response rate (ORR), disease control rate (DCR), and survival time) were evaluated and compared.

**Results:**

A total of 53 seed strands were successfully inserted and replaced in Group A, with a technical success rate of 100%. No procedure-related death or severe complications occurred in both group. Migration of seed strand or drainage tube was the most common complication. The Girignon grade of hydronephrosis was significantly improved 1, 3 and 6 months after procedure in both groups. DCR in Group A were 96.2%, 80.0%, and 70.0% at 1-, 3-, and 6-month follow up, respectively. At 1 and 6 months later, ORR in Group A were significantly higher than those in Group B (*p* < 0.05). The median overall survival were 30.0 months in Group A and 16.1 months in Group B, respectively (*p* = 0.04). The median progression-free survival were 11.1 months in Group A and 6.9 months in Group B, respectively (*p* = 0.09).

**Conclusion:**

Intraluminal Iodine-125 seed strand brachytherapy and percutaneous nephrostomy is safe and effective in patients with ureteral carcinoma, with higher ORR and median overall survival than patients underwent percutaneous nephrostomy without seed strand.

## Background

Ureter transitional cell carcinoma account for about 1–3% of urinary transitional cell carcinoma cases [1]. Surgical resection is the standard treatment for patients with resectable ureteral carcinoma, and radical ureterectomy with excision of the kidney and bladder cuff resection is generally recommended [2]. Unfortunately, radical resection causes the loss of kidney, which is not an easy choice for patients with renal insufficiency, isolated kidney or bilateral ureteropathy. Some patients do not undergo radical nephroureterectomy due to advanced age, comorbidities, or intolerance or refusal of surgery [3].

Traditional external radiotherapy is usually recommended for patients with advanced ureteral carcinoma; however, ureteral lesions often close to the intestines, spinal cord, and blood vessels, radiotherapy may result in intestinal injury, spinal cord damage, or retroperitoneal fibrosis [4]. As a novel kind of radiotherapy, intraluminal brachytherapy with radioactive iodine-125 seed strand has been used in some malignant tumors and shows encouraging efficacy [5], including advanced/recurrent esophageal cancer, malignant biliary obstruction, and hepatocellular carcinoma with portal vein tumor thrombus [6–8]. Inspired by previous intraluminal brachytherapy, we envision whether iodine-125 seed strand brachytherapy is also suitable for the treatment of malignant ureteral obstruction. We herein presented 48 patients with ureteral carcinoma who were treated with intraluminal iodine-125 seed strand brachytherapy and percutaneous nephrostomy.

## Methods

### Patients

**Fig. 1:**
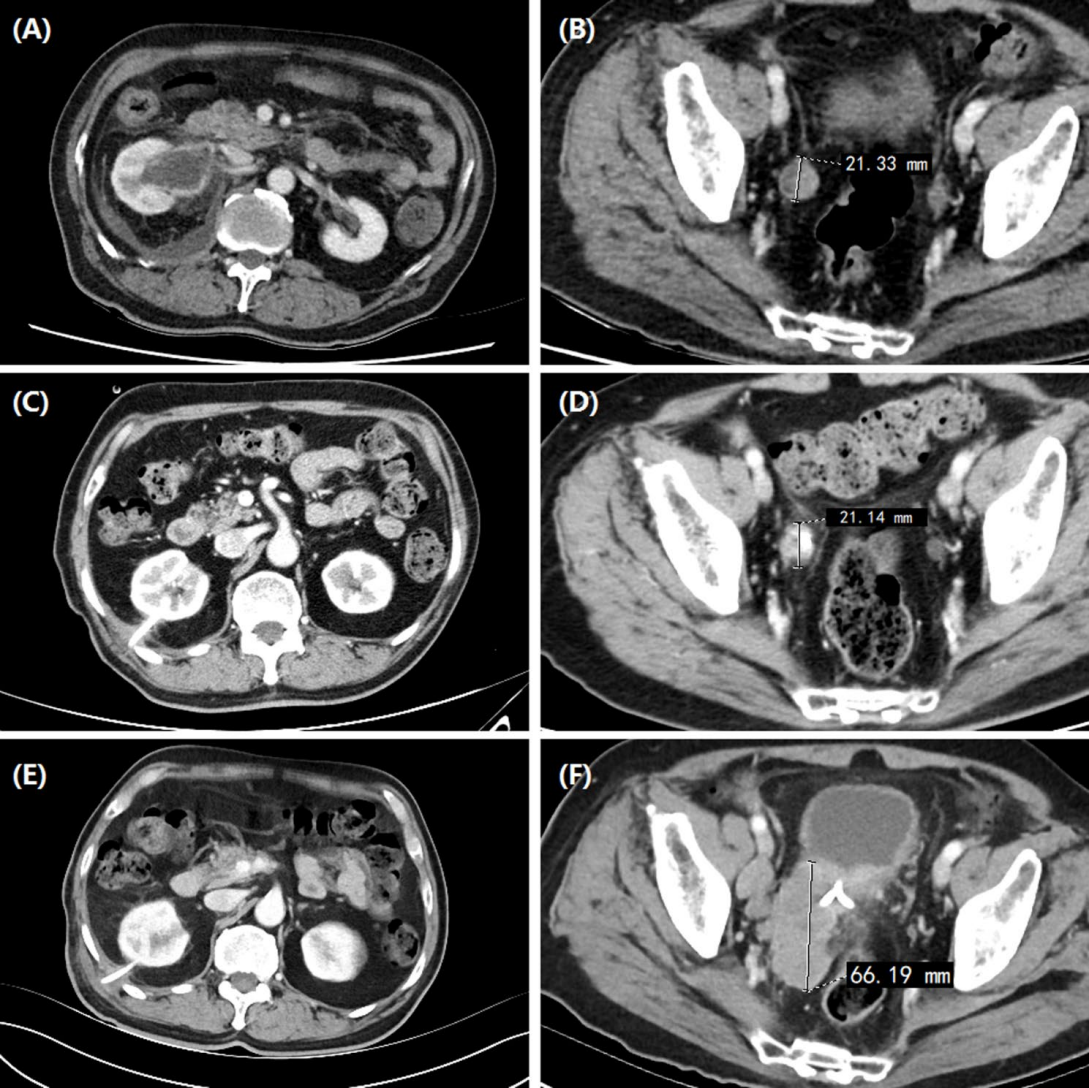
A 81-year-old male patient with right ureteral carcinoma. (**A**) The pretreatment CT showed obvious dilatation of the renal pelvis and hydronephrosis; (**B**) CT showed ureteral carcinoma (21.33 mm in diameter) in the lower segment of the ureter; (**C**-**D**) The posttreatment CT showed that the hydronephrosis disappeared after nephrostomy, and the tumor diameter was 21.14 mm about 3 months later; (**E**-**F**) The posttreatment CT showed PD after 4 years according to the tumor evaluation system;

From January 2014 to January 2023, 48 patients with ureteral carcinoma treated by intraluminal brachytherapy and/or percutaneous nephrostomy were enrolled. Iodine-125 seed strand was inserted in 26 patients under c-arm CT and fluoroscopic guidance (Group A), and 22 patients underwent percutaneous nephrostomy without seed strand (Group B). Contrast-enhanced computed tomography (CT) and/or magnetic resonance imaging (MRI) were carried out at baseline (Fig. 1AB; Fig. 3A). The inclusion criteria: Primary or recurrent ureteral carcinoma, or metastatic carcinoma of ureter; age > 18 years, with a estimated life expectancy exceeds 3 months; patients with renal insufficiency, isolated kidney or bilateral ureteropathy; patients not suitable radical nephroureterectomy due to advanced age, comorbidities; Patients with intolerance or refusal of surgical resection. Exclusion criteria: severe cardiovascular comorbidities including unstable angina, myocardial infarction or uncontrolled hypertension; coagulopathy or bleeding diathesis; severe infection; patients without follow up data. This retrospective study was conducted in accordance with the Declaration of Helsinki, and was approved by the institutional review board of the First Affiliated Hospital of Zhengzhou University (ethics number: 2014-ky-049). Informed consent had been obtained from all patients.

**Fig. 2:**
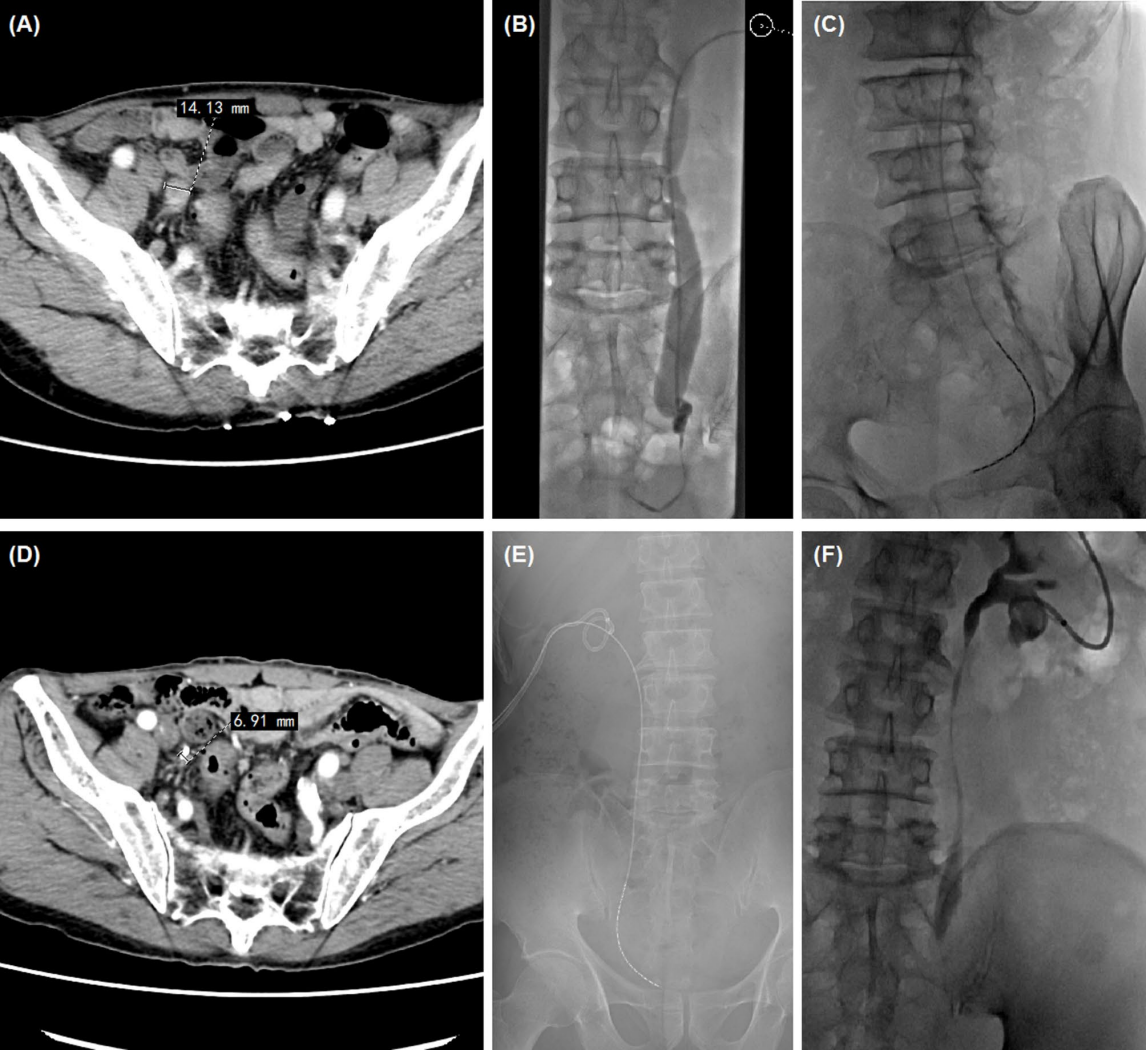
A 67-year male treated by iodine-125 seed strand for ureteral carcinoma. (**A**) CT revealed ureteral carcinoma in the right ureter, with a diameter of 14.13 mm. (**B**) Urothrography revealed a segmental occlusion in the lower segment of right ureter. (**C**) A Iodine-125 seed strand with 20 seeds was inserted. (**D**) CT revealed a significant shrunk tumor (6.91 mm in diameter) after 2.2 months’ follow-up. (**E**-**F**) The iodine-125 seed strand was removed after 6.8 months, and normal urothrography was shown

### Nephrostomy under c-arm CT and fluoroscopic guidance

**Fig. 3:**
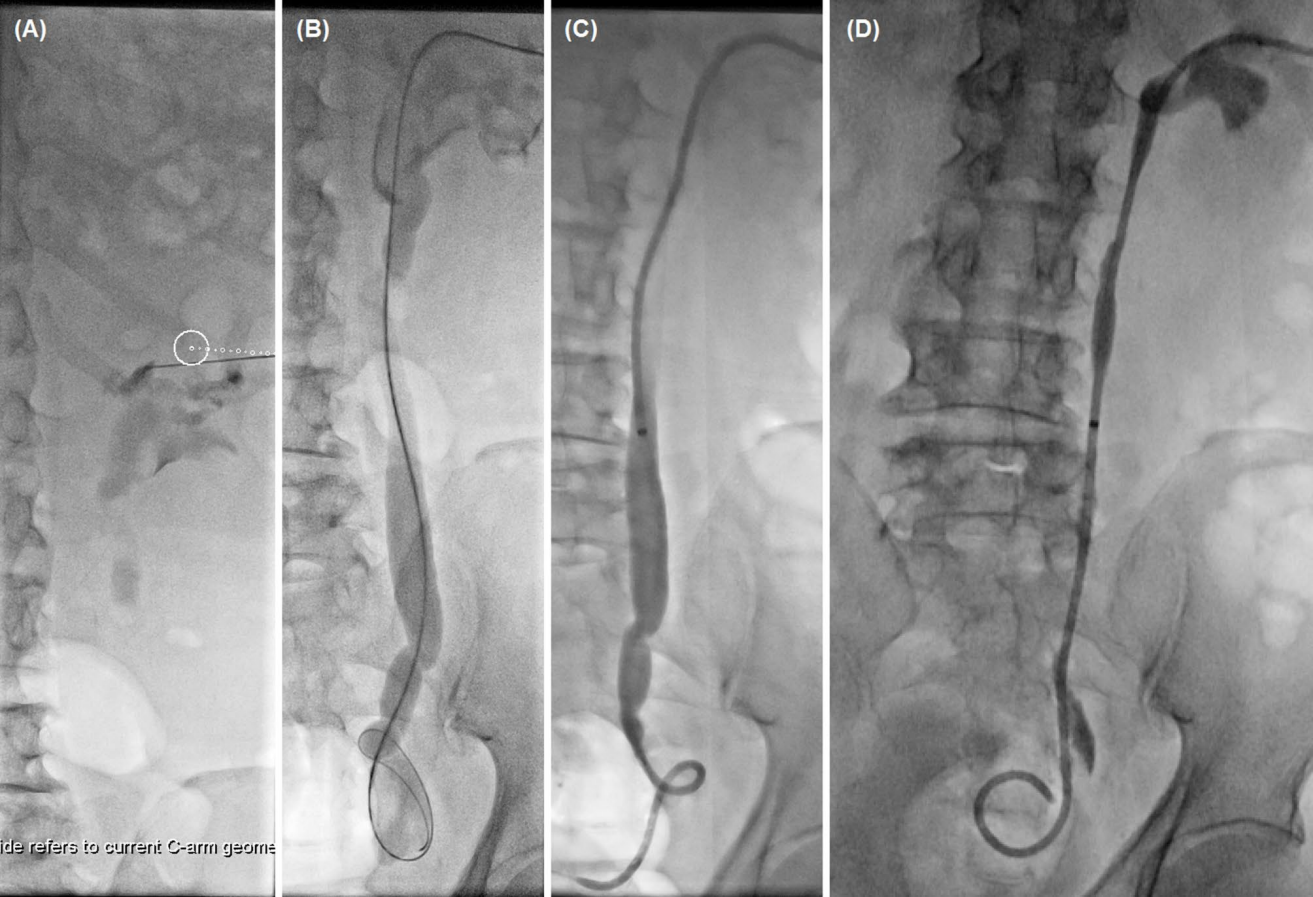
Percutaneous nephrostomy for 81-year male patient with right ureteral carcinoma. (**A**) Percutaneous renal pelvis puncture was performed under the virtual navigation of c-arm CT iGuide and fluoroscopic guidance. (**B**) A 0.035 inch guidewire and a 5 F catheter were passed through the ureteral occlusion segment. (**C**) A 8.5 F drainage tube was inserted for urine drainage. (**D**) Urothrography was performed again after 1.0 month

The patient was prone on the examination table and percutaneous renal pelvis puncture was performed under the virtual navigation of c-arm CT iGuide and fluoroscopic guidance. The upper segment of the renal pelvis and the ureter were visible by urography, and the involved ureter was shown as local stenosis or occlusion (Figs. 3 and 2B). A 0.035 inch guidewire and a 5 F catheter were passed through the ureteral stenosis or occlusion segment into the bladder. The lesion in the ureter was shown as filling defect of the contrast agent. The ureteral obstruction length was measured to assess the number of seeds placements. A stiff guide wire was exchanged and 8 F short sheath was inserted. Another stiff guide wire was introduced via the short sheath. After removal of short sheath, a 8.5 F drainage tube was inserted along a stiff guide wire into renal pelvis for urine drainage.

### Iodine-125 seed strand

**Fig. 4:**
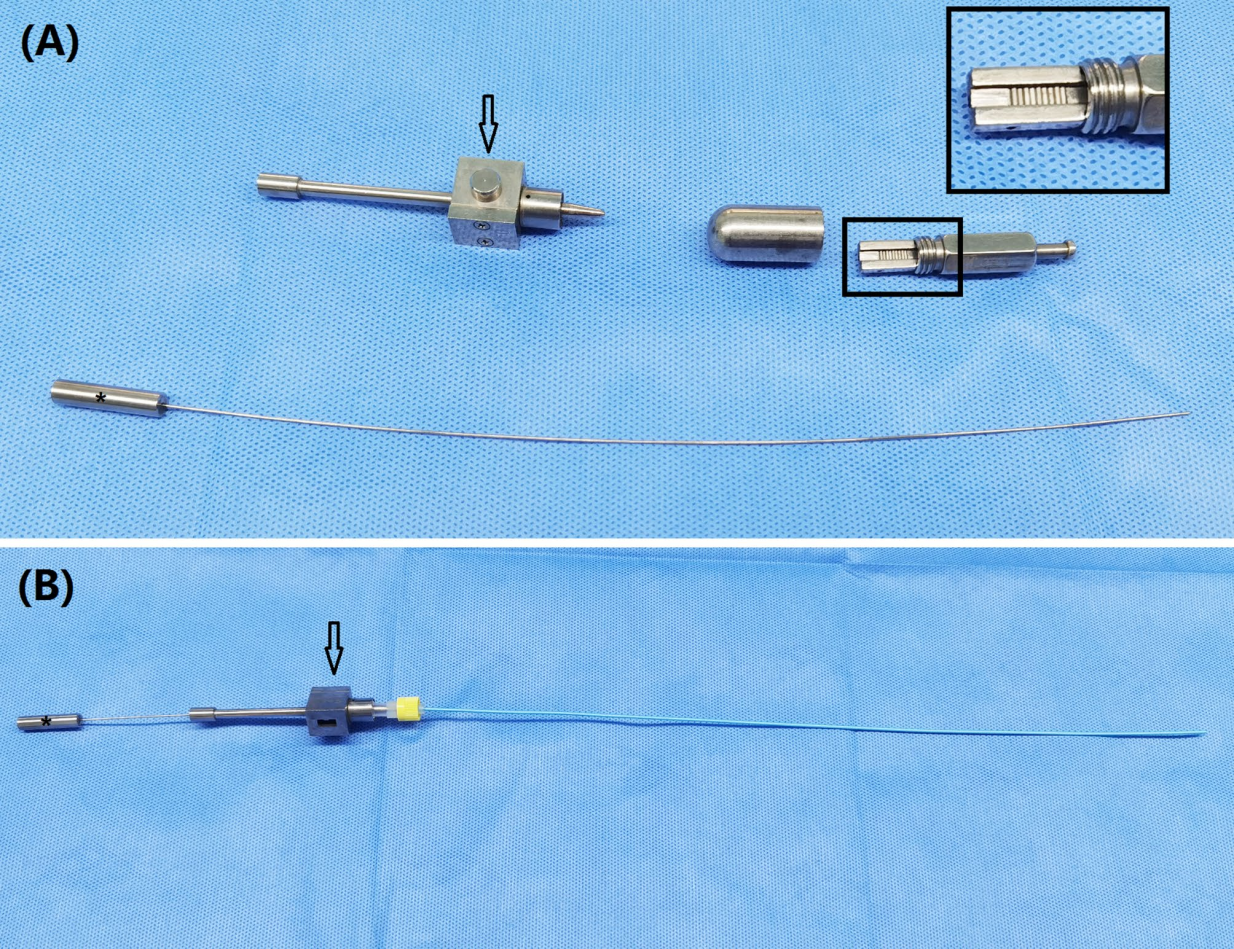
Iodine-125 seed strand. (**A**) A handspike (*) was used to push the Iodine-125 seeds in the seed implantation gun (arrow) into a 3 F catheter, arranging the seeds in a row within the catheter to form a seed strand (**B**)

The seed strand was introduced into the lesion segment along another stiff guide wire and fixed to avoid migration. The iodine-125 seeds (Saide Biological Technology Co., Ltd., Tianjin, China) are 0.8 mm in diameter and 4.8 mm in length, and have a half-life of 59.6 days and an irradiation distance of 17 mm. A handspike was used to push the seeds in the seed implantation gun into a 3 F catheter, arranging the seeds in a row within the catheter to form a seed strand (Fig. 4). The length of seed strand was 2 cm longer than the lesion segment (Fig. 2C). Both ends of the seed strand are sealed with fire to avoid seed migration. The nephrostomy tube and iodine-125 seed strand were fixed on the body surface to prevent displacement. The seed strand and the drainage tube were removed or exchanged 2 to 3 months after the insertion.

### Evaluation and follow-up

The clinical outcomes (technical success rate, tumor sizes, hydronephrosis Girignon grade, complications, objective response rate (ORR), disease control rate (DCR), and survival time) were evaluated and compared. Technical success rate was defined as the successful placement of nephrostomy and seed strand. Two to 3 months later, abdominal CT scan was performed to evaluate clinical efficacy (Figs. 1C-F and 2D-F). The tumor diameter refers to the largest diameter in the cross section of the ureteral tumor. Tumor evaluation was performed by using RECIST to evaluate the efficacy.

### Statistical analysis

Data of normal distribution are expressed as the mean ± standard deviation, and other data are expressed as the median (Interquartile range (IQR)) or count (%). Two-way ANOVA test was used to analyze the change of tumor diameter and Girignon score between Group A and Group B. Student t test and Fisher exact test were used for statistical analysis. Overall survival (OS) and progression-free survival (PFS) were calculated using the Kaplan-Meier method (Prism 5.0, GraphPad Software, Inc., SanDiego, CA). Differences were considered statistically significant at *p <* 0.05.

## Results

### Patient characteristics

From January 2014 to January 2023, 48 patients with ureteral cancer not suitable for surgical resection were enrolled in this study. There were 12 patients with advanced ureteral carcinoma in Group A, 10 cases were T3N0M0, 1 case was T3N1M0 and 1 case was T3N2M0. Recurrent ureteral carcinoma was present in 4 patients (15.4%) and in 10 patients (38.5%) had metastatic ureteral carcinoma due to pelvic malignancy in Group A. Ten patients (38.5%) complained of gross hematuria in Group A, with median duration of symptom of 6.0 months. Five patients (19.2%) in Group A and 6 patients (27.3%) in Group B received synchronous radiochemotherapy, respectively (*p* = 0.73). Baseline characteristics are shown in Table 1, with no significant difference between Group A and Group B.

**Table 1:** Baseline characteristics of enrolled patients

Variables	Group A	Group B	*p* value
Mean age, years	64.7 ± 11.9	62.1 ± 15.8	0.52
Male/Female	14/12	8/14	0.26
Course of disease, months	6.0 (2.2, 20.5)	6.0 (0.6, 12.0)	0.37
Lesion types			0.91
Advanced ureteral carcinoma	12 (46.2%)	13 (59.1%)	
Recurrent ureteral carcinoma	4 (15.4%)	2 (9.1%)	
Metastatic ureteral carcinoma	10 (38.5%)	7 (31.8%)	
Location of tumor			0.15
Upper segment	4 (115.4%)	7 (31.8%)	
Middle segment	3 (11.5%)	5 (22.7%)	
Lower segment	19 (73.1%)	10 (45.5%)	
Synchronous radiochemotherapy	5 (19.2%)	6 (27.3%)	0.73
Local metastasis	11 (42.3%)	9 (40.9%)	1.00
Distant metastasis	4 (15.4%)	6 (27.3%)	0.48
Gross hematuria	10 (38.5%)	6 (27.3%)	0.54
Comorbidities			0.08
Diabetes mellitus	5 (19.2%)	3 (13.6%)	
Coronary heart disease	5 (19.2%)	1 (4.5%)	
Old cerebral infarction	3 (11.5%)	1 (4.5%)	
Hypertension	10 (38.5%)	7 (31.8%)	

### Procedure outcomes of iodine-125 seed strand

As shown in Table 2, a total of 53 seed strand sessions were successfully inserted and replaced in Group A, with a technical success rate of 100%. Twelve patients (46.2%) completed 2 to 5 sessions of seed strand replacement or removal, and the median duration of renal drainage tube retention was 2.9 months (IQR 1.7–3.4). The median procedure time was 62 min in Group A and 45.0 min in Group B, respectively (*p* = < 0.01). A median of 20 (IQR 15.0-23.8) iodine-125 seeds was loaded in each seed strand. Twenty patients (76.9%) received other interventional treatments in Group A, including transarterial chemoembolization (TACE) (n = 10) and percutaneous seed implantation (n = 5), and both (n = 5) for progressive and migrated tumors.

**Table 2:** Clinical data of procedure and complications

Variables	Group A	Group B	*p* value
Procedure time, minutes	60.0 (45.3, 80.8)	45.0 (31.0, 60.0)	< 0.01
Duration of renal drainage tube retention, months	2.9 (1.7, 3.4)	3.0 (1.6, 5.0)	0.22
Number of seeds	20 (15.0, 23.8)	-	-
Median hospital stay, days	13.0 (8.0, 21.0)	13.0 (8.3, 25.8)	0.20
Median medical cost, ×10^4^	4.0 (2.5, 5.5)	3.4 (1.9, 7.0)	0.88
Complications, n (%)	11 (42.3%)	7 (31.8%)	0.56
Strand or tube migration	7 (26.9%)	3 (13.6%)	0.31
Mild pain	4 (15.4%)	4 (18.2%)	1.00
Obstruction of tube	1 (3.8%)	0 (0.0%)	1.00
Fever	2 (7.7%)	0 (0.0%)	0.49
Girignon score			> 0.05
Before procedure	3.9 ± 1.3	4.4 ± 0.9	
1 month later	1.4 ± 1.0*	2.4 ± 1.8*	
3 months later	1.3 ± 0.8*	2.2 ± 1.5*	
6 months later	1.5 ± 0.9*	2.3 ± 1.6*	
Lesion diameter, mm			
Before procedure	19.2 (15.8, 24.1)	25.0 (16.6, 45.9)	> 0.05
1 month later	13.0 (10.0, 19.4)	30.9 (12.5, 41.3)	< 0.01
3 months later	13.2 (10.3, 23.5)	27.4 (19.4, 36.6)	> 0.05
6 months later	14.0 (0.0, 21.5)	22.5 (15.0, 37.9)	> 0.05

*, *p* < 0.05 vs. before procedure

## Complications

There was no procedure-related death or severe complications in both groups. Minor complications were observed in 11 (42.3%) patients in Group A and 7 patients (31.8%) in Group B, respectively (*p* = 0.56). Migration of seed strand and drainage tube was observed in 7 patients (26.9%) and 3 patients (13.6%), and adjustment, replacement or removal of seed strand or drainage tube was performed. Mild pain during and after seed strand placement was observed in 4 patents (15.4%), and fever was observed in 2 patients (7.7%). Obstruction of nephrostomy tube was observed in one patient in Group A, and the tube was successfully replaced.

### Tumor response

The Girignon grade of hydronephrosis was significantly improved 1, 3 and 6 months after procedure in both groups. One patient (4.0%) and 3 patients (15.0%) achieved a complete response 3 and 6 months after seed strand placement, respectively, no complete response was observed in Group B. Partial response was observed in 9, 9 and 3 patients in Group A at 1-, 3-, and 6-month follow up. The ORR in Group A were 34.6%, 40.0% and 30.0% respectively at 1, 3, and 6 months. At 1 and 6 months later, ORR were significantly higher in Group A than those in Group B (*p* < 0.05). The DCR in Group A in Group A were 96.2%, 80.0%, and 70.0% respectively at 1, 3, and 6 months (Table 3).

**Table 3:** Treatment responses at 1, 3 and 6 months after procedure

Response	Group A			Group B		
	1 month	3 months	6 months	1 month	3 months	6 months
CR	0 (0.0%)	1 (4.0%)	3 (15.0%)	0 (0.0%)	0 (0.0%)	0 (0.0%)
PR	9 (34.6%)	9 (36.0%)	3 (15.0%)	2 (9.1%)	2 (10.5%)	2 (11.8%)
SD	16 (61.5%)	10 (40.0%)	8 (40.0%)	17 (77.3%)	10 (52.6%)	8 (47.1%)
PD	1 (3.8%)	5 (20.0%)	6 (30.0%)	3 (13.6%)	7 (36.8%)	7 (41.2%)
ORR	9 (34.6%)	10 (40.0%)	6 (30.0%)	2 (9.1%)*	2 (10.5%)*	2 (11.8%)
DCR	25 (96.2%)	20 (80.0%)	14 (70.0%)	19 (86.4%)	12 (63.2%)	10 (58.8%)

CR = Complete response, PR = Partial response, SD = Stable disease, PD = Progressive disease, ORR = Objective response rate, DCR = Disease control rate; *, *p* < 0.05

### Survival outcomes

Ten patients died of tumor progression and one died of cerebrovascular accident in Group A. The median overall survival were 30.0 months in Group A and 16.1 months in Group B, respectively (*p* = 0.04). The 6-, 12-, and 36-month OS in Group A were 96.0%, 86.3% and 49.3%, respectively. The 6-, 12-, and 36-month PFS were 69.4%, 47.3% and 10.8%, respectively. The median progression-free survival were 11.1 months in Group A and 6.9 months in Group B, respectively (*p* = 0.09).

## Discussion

Since the renal pelvis, ureter and bladder all originate from the mesoderm, with similar biological and morphology behavior, radical resection of bladder and ureteral carcinoma requires simultaneous resection of the bladder and ureter to reduce tumor metastasis and recurrence. A recurrence rate of 15–50% is observed in ureteral carcinoma after surgical resection of unilateral upper urinary carcinoma, and even reported a high rate of up to 70% [2, 9]. Radical nephrectomy is traumatic and costly, patients have to lose their kidneys, but the recurrence rate is still high. Some patients do not undergo radical nephroureterectomy due to advanced age, comorbidities, or intolerance or refusal of the procedure [3]. Besides, locoregional urothelial malignancies of urinary tract was treated by adjuvant radiotherapy or combined with chemotherapy [10–12], and confocal laser endomicroscopy has been used for upper tract urothelial carcinoma [13, 14].

Currently, cavity organs such as esophageal cancer have been treated with radioactive stent [15] and nutrient tube [6], and iodine-125 seeds strand have been used to treat malignant bile duct obstruction [16, 17] and portal vein tumor thrombus [18], and showed satisfactory clinical results. Inspired by previous intraluminal brachytherapy, we envision whether iodine-125 seed strand brachytherapy is also suitable for the treatment of malignant ureteral obstruction [19], considering that the ureter is a thin, long, and tubular shape organ. The iodine-125 seeds have a irradiation distance within 1.7 cm; intraluminal low-dose brachytherapy may theoretically play a safer and better role for ureteral carcinoma [19]. We herein presented 48 patients with ureteral carcinoma who were treated with intraluminal iodine-125 seed strand brachytherapy and percutaneous nephrostomy.

In this study, 53 seed strands were successfully inserted and replaced, with a technical success rate of 100% and no severe complications. The Girignon grade of hydronephrosis was significantly improved 1, 3 and 6 months after procedure in both groups. At 1 and 6 months later, ORR in Group A were significantly higher than those in Group B. The median overall survival in Group A was also significantly longer than those in Group B Our results suggest that intraluminal iodine-125 seed strand brachytherapy by and percutaneous nephrostomy may be more effective than patients underwent percutaneous nephrostomy without seed strand. Iodine-125 seed strand brachytherapy by and percutaneous nephrostomy can be alternative to patients who are not suitable for surgical resection.

Regarding safety, it is also reported that iodine-125 brachytherapy may cause radiation damage to vascular, nerve, and tracheal tissues [20–22], but the results all showed mild target and surrounding organ damage. In our study, no procedure-related death or severe complications occurred. Only 11 (42.3%) patients had minor complications, and migration of seed strand was the most common complication. Besides, as the ureteral tumor grows, it may lead to narrowing of the ureterial lumen and eventually occlusion. Urography presented with local occlusion of the ureter without contrast. However, there is still potential lumen and tiny gaps in the occlusion segment of ureter. After repeated attempts, it is possible for the catheter and the guide wire to pass through the occlusion segment. Of course, not all occluded segments can pass. If the tumor is severely invaded, the guidewire may not pass through the occluded segment of ureter. In this study, all occluded segments were passed successfully without severe complication, such as massive bleeding or perforation of the ureter.

There were several limitations in this study: (1) This was a single-center, retrospective study, further studies with larger sample size are required. (2) About half of patients received only one session of seed strand placement, which may contribute to confounding factor affecting efficacy evaluation. (3) The effective radiation distance of the seed is short, and it may not be enough for metastatic cancer or large lesions, other local or systematic treatment are required.

In conclusion, intraluminal Iodine-125 seed strand brachytherapy and percutaneous nephrostomy is safe and effective in patients with ureteral carcinoma, with higher ORR and median overall survival than patients underwent percutaneous nephrostomy without seed strand.

## Data Availability

The datasets generated during and/or analysed during the current study are available from the corresponding author on reasonable request.
